# Performance Study of the Application of Artificial Neural Networks to the Completion and Prediction of Data Retrieved by Underwater Sensors

**DOI:** 10.3390/s120201468

**Published:** 2012-02-02

**Authors:** Carlos Baladrón, Javier M. Aguiar, Lorena Calavia, Belén Carro, Antonio Sánchez-Esguevillas, Luis Hernández

**Affiliations:** 1 Universidad de Valladolid, Dpto. TSyCeIT, ETSIT, Paseo de Belén 15, 47011 Valladolid, Spain; E-Mails: javagu@tel.uva.es (J.M.A.); lcaldom@ribera.tel.uva.es (L.C.); belcar@tel.uva.es (B.C.); antsan@tel.uva.es (A.S.-E.); 2 CIEMAT, Autovía de Navarra A15, salida 56, Lubia, 42290 Soria, Spain; E-Mail: luis.hernandez@ciemat.es

**Keywords:** artificial intelligence, artificial neural networks, data completion, data prediction, underwater sensors

## Abstract

This paper presents a proposal for an Artificial Neural Network (ANN)-based architecture for completion and prediction of data retrieved by underwater sensors. Due to the specific conditions under which these sensors operate, it is not uncommon for them to fail, and maintenance operations are difficult and costly. Therefore, completion and prediction of the missing data can greatly improve the quality of the underwater datasets. A performance study using real data is presented to validate the approach, concluding that the proposed architecture is able to provide very low errors. The numbers show as well that the solution is especially suitable for cases where large portions of data are missing, while in situations where the missing values are isolated the improvement over other simple interpolation methods is limited.

## 1. Introduction

Underwater sensors are always subject to a specific set of limitations not encountered by surface and earth based devices. Firstly, they are inherently difficult to reach, making maintenance operations expensive and at times impossible. Accordingly, the first result is that these sensors might have to optimize energy consumption [1], communicating and reporting less often and with less power than desirable in principle [2], in order to save batteries that might never be replaced. Additionally, the hostile conditions of rivers, seas and oceans, which include variable currents, animal attacks, corrosion, *etc*., might easily cause temporal or permanent malfunctions that will result in non trustable measures, or in complete breakdown of the sensor [3], that cannot be easily repaired or substituted.

Due to these specific circumstances, information retrieved from underwater sensors is often less than perfect, with missing readings or devices reporting with not enough frequency. One simple example of this fact is the data about a set of ocean magnitudes (temperature, salinity or density) offered by GoMOOS (Gulf of Maine Ocean Observing System—http://www.gomoos.org/gnd/). While surface readings have a period of thirty minutes, underwater sensors report only each hour, and it is not extremely uncommon to find void or wrong readings. Specifically, large portions of data are found missing for different magnitude readings during long periods of time, presumably because that sensor was failing and maintenance cannot be conducted immediately due to time and money constraints. This kind of massive failures can happen for periods as long as six months.

For instance, for the specific case of data retrieved by the “Jordan Basin” node between 31 August 2008 and 5 March 2010, with surface sensors normally reporting each half an hour and underwater sensors reporting hourly, a total of 26,448 reports are expected. From those, only 24,663 (93.25%) were received and 13,088 (49.48%) include underwater data. But even more, as each report is comprised by the readings of 23 independent sensors (for different magnitudes at different depths), the number of complete reports received, in which none of those sensors failed and the 23 readings were received, is only of 5,108, that is, 39.02% of the reports received containing underwater data, and only the 19.31% of the expected reports.

Applications depending on these (and other similar) datasets [4–7] may therefore experience a significant drop in their efficiency with respect to the ideal case in which the information is reported consistently. For some of these applications, such as environmental studies, this may not be extremely critical, but for others, for instance seismic vigilance and monitoring, it may even have an impact on human lives. In any case all applications may benefit from a better set of data. In all cases, the performance of the applications over the underwater data drops ranging from catastrophic failures (the algorithms depend on the data to be complete in order to work) to reduced efficiency when confronted with incomplete data. In most cases, the gaps in the dataset have to be filled with, at least, a simple interpolation method in order for the specific application to work.

The aim of this work is to mitigate this data incompleteness problem by employing Artificial Intelligence (AI) techniques (and specifically Artificial Neural Networks—ANN) to improve the quality of data retrieved by underwater sensors. While the literature is rich in applications of this kind of algorithms for underwater vision and sonar [8–10], it is quite scarce when it comes to other underwater parameters [11–15]. While many AI techniques and algorithms are available and documented for this kind of data processing [16–20], ANN are a very popular method, with many easy to find implementations, and what is more important, easy to operate at a high level (even with user-friendly graphical interfaces) by a non-expert in the field.

The strategy adopted along this work is to use known data, that is, the information properly reported by the sensors, to extract the missing data due to, for instance, a malfunction in a sensor, or a sensor not reporting in order to save energy. There is a basic assumption supporting this approach: that the different variables (the different magnitudes monitored at different depths and locations) are correlated. In the case of underwater sensors, magnitudes such as temperature, salinity, visibility and density are physically related one to the other, and due to proximity, they are also related to nearby values in depth and location. Under these conditions it is possible to infer and extract an approximate of the missing information from the existing data.

Therefore, the aim of this work is to evaluate the performance of ANNs (a fairly generic computing paradigm suitable for a wide variety of domains, from climate prediction to image pattern recognition) when applied to completion and prediction of underwater magnitudes, determining if they are a suitable approach to improve the quality of historical and real-time underwater datasets and quantify the kind of precision that can be expected from the predictions.

After this Introduction, the paper presents in Section 2 the dataset employed for the study, and Section 3 the different Neural Network architectures designed for data inference and prediction. Afterwards, Section 4 exposes and analyses the tests conducted and the experimental results obtained. Finally, Section 5 summarizes the conclusions of this work.

## 2. Underwater Sensor Dataset

In order to perform the study presented in the Introduction, a dataset containing information retrieved by underwater sensor networks has to be employed. The underwater database provided by GoMOOS, that will soon be taken over by NERACOOS (Northeast Regional Association for Coastal and Ocean Observing Systems), has been selected for this purpose due to the wide array of sensors provided, a large amount of different locations monitored, several different magnitudes and depths scanned, long historical data, and easy Web based interface to access the information.

Along this work, mainly two sets of data from the GoMOOS database have been employed. One of them is the historical data for the NERACOOS “Jordan Basin” sensor buoy, spanning from 31 August 2008 to 5 March 2010, which reported hourly visibility at the surface and salinity, density and temperature measures at depths of 1 m, 20 m, 50 m, 100 m, 150 m, 200 m and 250 m (with an additional temperature measure at 2 m depth), for a total of 23 values per hourly report. The second one is the historical data for the NERACOOS “Northeast Channel” sensor buoy, spanning from 31 August 2008 to 30 November 2009, which reported hourly visibility at the surface and salinity, density and temperature at depths of 1 m, 20 m, 50 m, 100 m, 150 m and 180 m (again, with an additional temperature measure at 2 m depth), summing 20 values per hourly report. These two locations were selected as they offered the most complete historical data from all the sensors of the system.

A total of 5,108 and 2,045 “clean” reports (reports which presented all their 23/20 readings with proper values) have been identified and employed in the study from the “Jordan Basin” and “Northeast Channel” historical data, respectively. As discussed in the Introduction, many other reports were generated by both of them, but were not usable in the study due to the lack of one or more readings.

## 3. Neural Network Architecture

Within the general domain of improving the quality of data retrieved by underwater sensors, there are different specific approaches that can be followed and consequently, different architectures for the Neural Networks designed to implement them.

Within the scope of this work, two different scenarios are considered: the first one has been named *data completion*. In this scenario, a report of a series of *n* sensors is received for a time *t*, but the information from the *n*-th sensor is missing. The aim is to infer the reading from that sensor, at time *t*, using the available readings from the other *n* − 1, at time *t* as well. To make it possible, the Neural Network is trained with the complete reports received at times before *t*.

Three different approaches are used to cope with the *data completion* scenario. In the first one, sensors are monitoring the same magnitude at different depths, taking advantage of the correlation among different values of the same magnitude at different depths. In the second one, sensors are monitoring different magnitudes at the same depth, taking advantage of the correlation among temperature, density, salinity, *etc*. In the third one, sensors are monitoring the same magnitude, at the same depth, but in different locations, and predictions are made on the basis of the correlation in the magnitude values among places which are close one to the other.

The second scenario considered has been named *data prediction*. In this scenario, a sensor monitoring a specific magnitude at a specific depth sends reports periodically. The problem here is, at time *t*, to predict which readings will be reported in the future moments *t* + 1, *t* + 2, *etc*., taking into account readings before time *t*.

Artificial Neural Networks [21–23] are an extremely distributed computing paradigm suitable for a variety of AI-like operations [24–32], including clustering, pattern recognition, data prediction, *etc*. This computing paradigm is rather generic, meaning that its algorithms (the architectures of the neural network) are fundamentally the same across different data domains (weather, health, *etc.*), and only depend on the kind of operation to be performed (prediction, fitting, pattern recognition, *etc*.) and the shape of the data (with a set of parameters, like the size of the input/output stages) [33–37]. Therefore, for this work, a specific architecture has to be chosen, and the parameters of the network tuned to the shape of the data available.

The basic idea behind a neural network is that a set of independent neurons are linked together. Neurons are excited through this links by the input values and by other neurons, propagating the excitation towards an output stage, which generates an output. During the training stage, the network is presented with a set of input-output pairs, and the weights of the links among the neurons are modified in a way that each input produces the expected output. Afterwards, in operation mode, the weights are fixed and the network throws an output when confronted with each new input.

**Figure 1.**
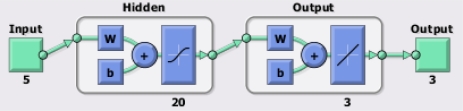
Basic scheme of the proposed neural network as shown by MATLAB.

For the two scenarios considered along this work, a specific kind of neural network, known as *fitting neural network,* has been employed. This kind of neural network presents an input layer, an output layer and a hidden layer in-between. In our proposed architecture, the hidden layer has a fixed size of 20 neurons (a number capable of handling complex data), while the input and output layers are adjusted to the size of the input and output vectors of the different scenarios. For instance, Figure 1 presents the basic scheme of the proposed neural network for a case with a five-component input vector and a three-component output vector: completion of the three deepest temperature values for a buoy (output) using the other five known values closer to the surface (input). The backpropagation *Levenberg-Marquardt* [38] training algorithm has been employed. All networks have been implemented and tested using MATLAB.

## 4. Experimental Results

For all the experiments conducted, the way in which the neural network operates is similar. 60% of the data available is used as training data: the network is configured in training mode, and pairs of input vectors with their corresponding known output vectors are presented to it, so as the network can internally modify the weights of the links to provide the desired outputs. Then, the other 40% of the available data is used as test data: the network, configured in operation mode, is presented with each input value to produce an output value. Finally, this output value is compared to the actual “target” value (according to the dataset) to determine the error between the expected output (the target value) and the actual output of the network.

### 4.1. Data Completion

For the first approach of the data completion scenario (in which a reading is predicted using other readings at different depths), the neural network has been employed over the data of two different locations (Jordan Basin and Northeast Channel) for the magnitudes Density, Temperature and Salinity. For each location and magnitude, four tests are conducted. In the first one, from all the readings at different depths, the deepest one is supposed missing and is predicted; in the second and third ones, the two and three deepest ones are considered missing and are predicted, respectively. In the fourth test, all underwater readings are predicted on the sole basis of the surface (1 m depth) reading.

For each test, the mean error of the prediction is calculated. The tests are run five times and the error results averaged accordingly to reduce the influence of the random initialization of the training algorithms. As a means of validation of the results, the error that would result when simply using the reading of the sensor at the nearest depth as a prediction of the missing reading/s has also been calculated.

**Figure 2.**
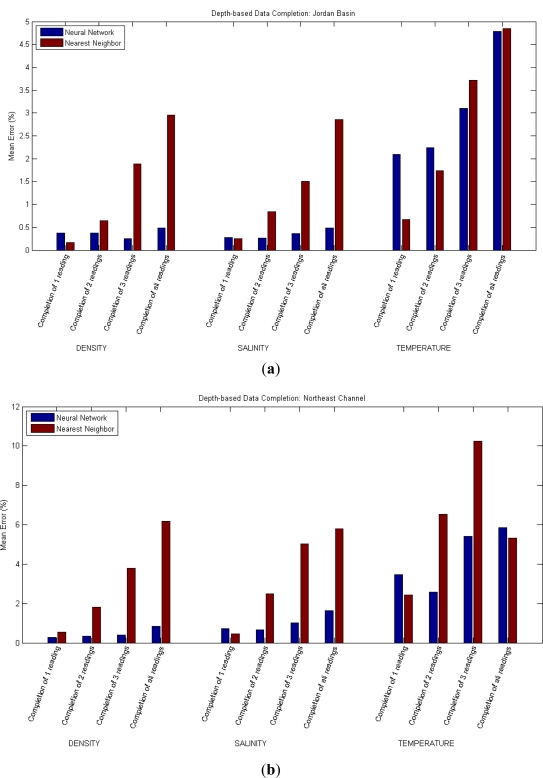
Results of Depth-based Data Completion: a reading is predicted using other readings of the same magnitude at different depths. (**a**) In Jordan Basin; (**b**) In Northeast Channel.

The mean errors resulting from the application of the neural network and from the usage of the nearest reading are presented and compared in Table 1 and Figure 2. Cases in which the simple prediction method performs better than the neural network are highlighted in red.

For the second approach of the data completion scenario (in which a reading is predicted using the readings of other magnitudes at the same location and depth) three tests have been conducted. In the first one, Density has been predicted on the basis of Salinity and Temperature; in the second and third ones, Salinity and Temperature are predicted, respectively, using the other two magnitudes.

**Figure 3.**
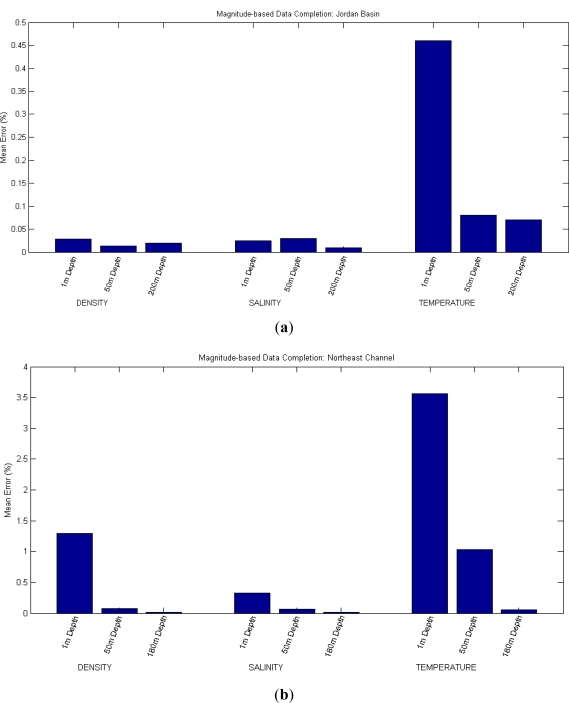
Results of Magnitude-based Data Completion: a reading is predicted using other readings of different magnitudes. (**a**) In Jordan Basin; (**b**) In Northeast Channel.

These tests are repeated for different depths and locations. All of them are again run for five times and the results averaged, giving the mean errors presented in Table 2 and Figure 3. In this case it is not possible to employ a simple prediction method using the nearest neighbor like in the first approach, because the inputs are magnitudes different from the output.

For the third approach of the data completion scenario (in which a value is predicted using the reading of the same magnitude, at the same depth, but on a different location) the readings at the Northeast Channel location have been used as an input to predict readings of the same magnitude, at the same depth, at the Jordan Basin location. In this approach, the error resulting from directly using the input value at Northeast Channel as the prediction of the value in Jordan Basin has also been calculated.

**Figure 4.**
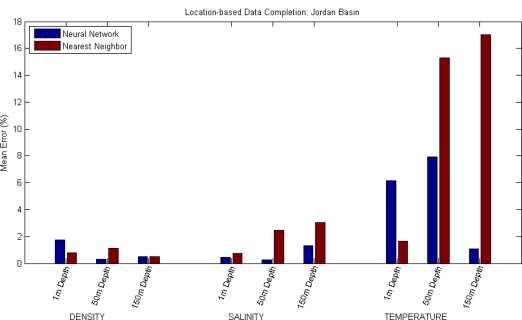
Results of location-based Data Completion in Jordan Basin: a reading is predicted using other readings of the same magnitude at different, nearby locations (Northeast Channel).

The tests have been conducted for the three magnitudes at three different depths, again five times each, and the resulting mean errors shown in Table 3 and Figure 4.

### 4.2. Data Prediction

**Figure 5.**
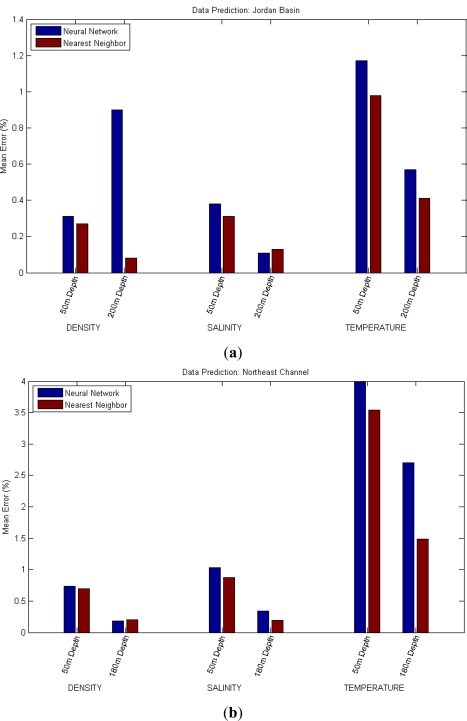
Results of Data Prediction: a reading is predicted using other readings at past times. (**a**) In Jordan Basin; (**b**) In Northeast Channel.

For the data prediction scenario, the idea is to predict one specific reading in a future moment *t* using past measures of the same magnitude at the same location and depth. Tests have been executed for the three magnitudes considered (Density, Salinity and Temperature) at different depths and locations. In this specific case, the measure predicted is *t* + 24 hours using the six last hourly readings as an input, and each test was again run five times, with the averaged mean errors represented in Table 4 and Figure 5. Again as a method of comparison, a simpler method has been tested using the last known value of the magnitude directly as the prediction, and tests in which this prediction was better than the one calculated by the neural network have been, as usual, marked in red.

Different tests (with different values of the time horizon to be predicted and the number of past readings considered) have been conducted, but the results yielded have been similar to the ones shown in Table 4, with little to no significant differences regarding their interpretation.

It is worth noting as well that when predicting a value for a given magnitude, depth and location, and the same value for the previous hour is available, simply using that value as a prediction normally yields extremely accurate results, with errors around 0.001%, due to the slow variations experienced by the monitored magnitudes.

### 4.3. Interpretation of the Results

The first obvious conclusion that can be extracted from a first glance analysis of the results is that the Data Prediction scenario is not too favorable for the neural network approach. Normally, variations of the values are slow and with a relatively small variance, which results in a good approximation of the future value simply by using an old measure of the same parameter. The second easy conclusion is that when the missing data is isolated, a simple prediction directly using the value from the previous hour is more often than not the best solution.

However in the majority of cases there are long portions of data missing or faulty. These errors usually come from broken sensors that need repairing and maintenance tasks that are generally costly due to the underwater situation of the sensors, and in this case using the neural network can potentially save money and time while keeping the quality of the data. Under this assumption, for the Data Completion scenario, it is easy to see that the first and third approaches (the ones based on inputs at different depths and locations respectively) yield good results in general, achieving errors ten times smaller than with a simple prediction based on the nearest neighbor (in depth or location). There are specific cases (when the nearest neighbor in depth or location is very near), however, where this simple prediction offers similar, and even better, results. The conclusion here is that it is normally useful to employ the neural network, but it is necessary to analyze the specific conditions of the data to be completed or predicted, because it could happen that the increased complexity of the neural network might not result always in a better performance.

However, it is on the second approach of the data completion scenario, the one in which completion is based on other magnitudes at the same location, time and depth, where the results of the neural network become extremely significant. In this context, the mean errors are extremely small, reaching even 0.01% for some situations. The straightforward conclusion is that the neural network proposed is extremely useful when a specific sensor for a magnitude is failing, but readings for other magnitudes at the same point are available.

In cases where the entire sensor array for a given location and depth is failing, and there are no readings for any magnitude there, it is then useful in the majority of cases, as mentioned earlier, to employ readings for the same magnitude at nearby depths and locations. But in this case it is necessary to specifically study the conditions, and how far those depths and locations are.

## 5. Conclusions

This paper presents an Artificial Neural Network solution for the completion and prediction of data retrieved by underwater sensors. This ANN has been applied to a set of real data extracted from the GoMOOS database to validate the approach under real conditions. The analysis of the results show that in these environments, while there are situations where simpler approaches are suitable for both tasks (especially when there are isolated readings of missing data), in the majority of cases the Neural Network shows a good performance, with very small mean errors. This is especially true in the cases where there are long time failures and big portions of missing data.

This is directly applicable in numerous situations related to underwater sensors. These sensors are normally deployed in hostile surroundings and are extremely difficult and expensive to reach for maintenance operations, so sensor failures are not uncommon, and when they happen, it is very difficult and takes a lot of time to repair or replace them (if it is possible at all). This results in large portions of data missing until these maintenance operations are carried out, and it is specifically in this situations when the Neural Networks proposed in this work show their full potential, because they are able to keep the quality of the data at near-zero cost, saving money from the costly maintenance operations, or during the time that those operations are performed.

The main conclusion is that the work presented along this paper can contribute to the improvement of the various underwater datasets available, incrementing the amount of valid and complete data that can be extracted from them. Due to the specific conditions of underwater sensors, as previously mentioned, the rate of complete data is usually low in this environment. Therefore, applications taking advantage of these datasets, and also those operating in real time with underwater data, can greatly benefit from the approach presented in this paper.

## Figures and Tables

**Table 1.** Results of Depth-based Data Completion: a reading is predicted using other readings of the same magnitude at different depths. The table shows mean errors for each case. In red, situations where the Nearest Neighbor completion performs better than the AAN.

**Location**	**Magnitude**	**Completion of 1 reading**		**Completion of 2 readings**		**Completion of 3 readings**		**Completion of all underwater readings**	

		**ANN**	**Nearest Neighbor**	**ANN**	**Nearest Neighbor**	**ANN**	**Nearest Neighbor**	**ANN**	**Nearest Neighbor**
Jordan Basin	Density	0.38%	0.17%	0.38%	0.64%	0.25%	1.89%	0.48%	2.96%
	Salinity	0.28%	0.25%	0.27%	0.84%	0.36%	1.51%	0.49%	2.86%
	Temperature	2.10%	0.67%	2.24%	1.74%	3.10%	3.72%	4.78%	4.85%

Northeast Channel	Density	0.28%	0.55%	0.33%	1.8%	0.39%	3.78%	0.84%	6.17%
	Salinity	0.71%	0.47%	0.65%	2.48%	1.02%	5.03%	1.63%	5.80%
	Temperature	3.47%	2.43%	2.57%	6.54%	5.42%	10.24%	5.84%	5.31%

**Table 2.** Results of Magnitude-based Data Completion: a reading is predicted using other readings of different magnitudes. The table shows mean errors for each case.

**Location**	**Depth**	**Completion of Density**	**Completion of Salinity**	**Completion of Temperature**
Jordan Basin	1 m	0.029%	0.025%	0.46%
	50 m	0.013%	0.03%	0.08%
	200 m	0.02%	0.010%	0.07%

Northeast Channel	1 m	1.29%	0.33%	3.56%
	50 m	0.08%	0.07%	1.03%
	180 m	0.011%	0.018%	0.06%

**Table 3.** Results of location-based Data Completion: a reading is predicted using other readings of the same magnitude at different, nearby locations. The table shows mean errors for each case. In red, situations where the Nearest Neighbor completion performs better than the AAN.

**Depth**	**Density**		**Salinity**		**Temperature**	

	**ANN**	**Nearest Neighbor**	**ANN**	**Nearest Neighbor**	**ANN**	**Nearest Neighbor**
1 m	1.74%	0.78%	0.45%	0.73%	6.17%	1.66%
50 m	0.30%	1.13%	0.27%	2.47%	7.90%	15.31%
150 m	0.48%	0.51%	1.30%	3.04%	1.09%	17.00%

**Table 4.** Data Prediction results: a reading is predicted using other readings at past times. The table shows mean errors for each case. In red, situations where the Nearest Neighbor completion performs better than the AAN.

**Location**	**Depth**	**Prediction of Density**		**Prediction of Salinity**		**Prediction of Temperature**	

		**ANN**	**Nearest Neighbor**	**ANN**	**Nearest Neighbor**	**ANN**	**Nearest Neighbor**
Jordan Basin	50 m	0.31%	0.27%	0.38%	0.31%	1.17%	0.98%
	200 m	0.09%	0.08%	0.11%	0.13%	0.57%	0.41%

Northeast Channel	50 m	0.74%	0.70%	1.03%	0.87%	3.99%	3.54%
	180 m	0.18%	0.20%	0.34%	0.19%	2.70%	1.49%
